# Spontaneous Heterotopic Pregnancy: Case Report and Literature Review

**DOI:** 10.3390/medicina56080365

**Published:** 2020-07-22

**Authors:** Miglė Černiauskaitė, Brigita Vaigauskaitė, Diana Ramašauskaitė, Mindaugas Šilkūnas

**Affiliations:** 1 Faculty of Medicine, Vilnius University, M. K. Čiurlionio g. 21, 03101 Vilnius, Lithuania; 2 Institute of Clinical Medicine, Faculty of Medicine, Vilnius University Clinic of Obstetrics and Gynaecology, Vilnius University Hospital Santaros Clinics, Santariškių g. 2, 08661 Vilnius, Lithuania; brigitavaig@gmail.com (B.V.); Diana.Ramasauskaite@santa.lt (D.R.); mindaugas.silkunas@gmail.com (M.Š.)

**Keywords:** heterotopic pregnancy, spontaneous pregnancy, ectopic pregnancy, laparoscopic salpingotomy

## Abstract

Heterotopic pregnancy is defined as a condition when intrauterine and extrauterine pregnancy occur simultaneously. It is a life-threatening condition that requires immediate and accurate diagnostics and treatment. We present a case of a 28-year-old primigravida female who conceived spontaneously and at her seventh week of gestation and was presented to the emergency department with weakness and acute pain in lower abdomen. Laboratory tests and transvaginal ultrasonography revealed the diagnosis of heterotopic pregnancy. Urgent laparoscopic salpingotomy was chosen as a treatment option. The ectopic pregnancy was successfully removed with the preservation of the intrauterine embryo and fallopian tubes. The course of pregnancy after the surgery was without complications, and a healthy baby was delivered at the 39th week of gestation. When treated properly and on time, a heterotopic pregnancy can result in live childbirth with favorable outcomes for both the child and the mother.

## 1. Introduction

Heterotopic pregnancy (HP) was first described in 1708 by Duverney. It is defined as a rare condition when intrauterine and extrauterine gestations coexist. The theoretically calculated incidence of a spontaneous HP is approximately 1 in 30,000 [1], while some risk factors increase the frequency up to 1 in 100 [2,3,4]. HP is a life-threatening condition that may lead to complications such as the rupture of an ectopic pregnancy (EP) and the loss of the intrauterine (IU) embryo after treatment [5].

We report a case of a spontaneous HP in a 28-year-old patient with the successful treatment of an EP with the preservation of fallopian tubes and the IU embryo, which resulted in a spontaneous delivery.

## 2. Case Presentation

**Figure 1:**
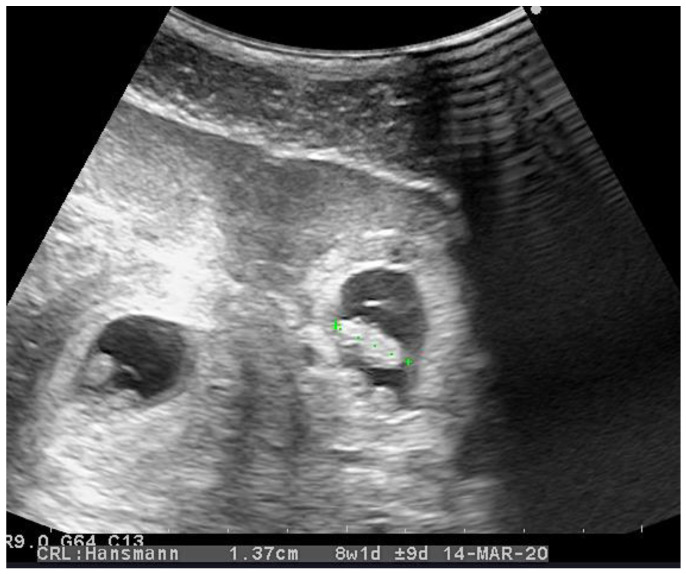
Transvaginal ultrasonography image of the uterus (transverse view) showing an intrauterine gestation (**left**) coexisting with an ectopic tubal pregnancy (**right**) with a sac containing an embryo with a crown rump length of 13.7 mm.

**Figure 2:**
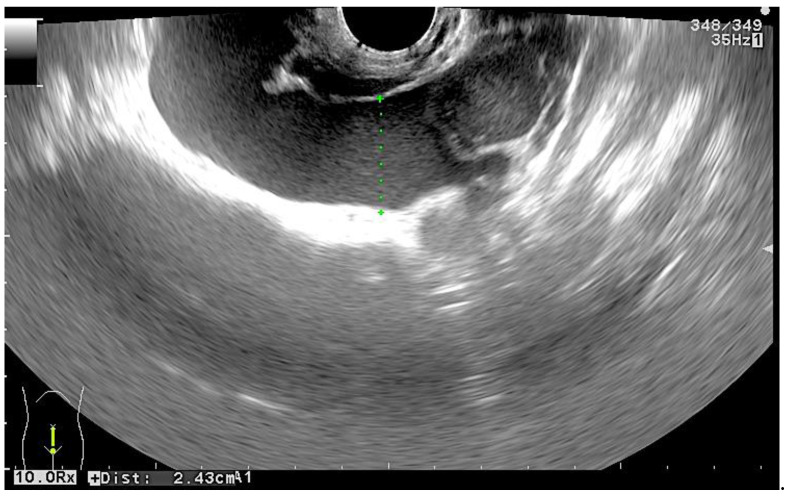
Transvaginal ultrasonography image showing free intraperitoneal fluid in pelvis.

**Figure 3:**
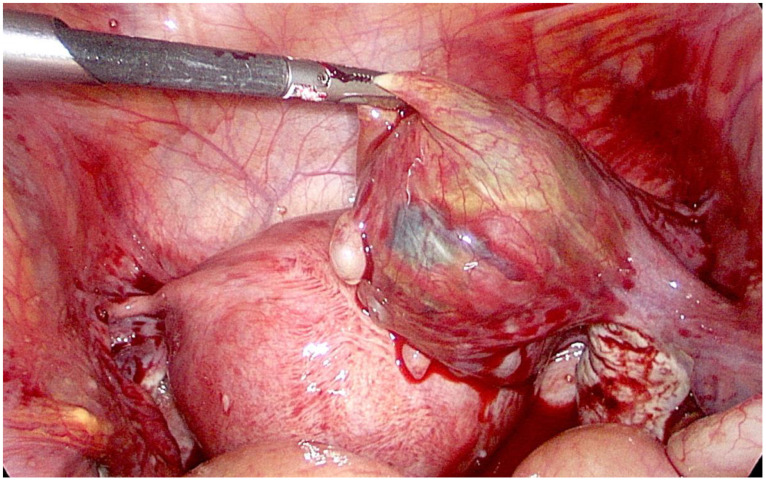
Laparoscopic findings of the ectopic pregnancy in the ampulla of the right fallopian tube with hemoperitoneum.

A twenty-eight-year-old otherwise healthy female (spontaneous pregnancy, gravida 1 para 0) at her seventh week of gestation was presented to the emergency department complaining of weakness and acute pain in lower abdomen and epigastric region. The pain had lasted for one day without nausea, vomiting, or other gastrointestinal symptoms. The patient did not have any vaginal bleeding and denied any other illnesses or allergies. A physical examination revealed a normal body temperature and arterial blood pressure (120/80 mmHg), along with tachycardia (94 beats per minute) and pain of the lower abdomen during palpation. Laboratory testing on admission showed an elevated white blood cell count up to 18.11 × 10^9^/L, a hematocrit of 0.285 L/L, and a serum hemoglobin concentration of 98 g/L, along with a normal blood platelet level of 329 × 10^9^/L. The serum β-human chorionic gonadotropin (β-hCG) level was 75,635 U/L. Transvaginal ultrasonography (TVUS) revealed intrauterine (IU) gestation with a sac of 25.2 mm in diameter and a crown–rump length (CRL) of 11.2 mm with a positive embryo heart rate and an extrauterine gestation in the right fallopian tube with a sac of 20.2 mm and a CRL of 13.7 mm with cardiac activity (Figure 1). TVUS also demonstrated free intraperitoneal fluid in the lesser pelvis (Figure 2). An urgent right laparoscopic salpingotomy was performed under general anesthesia, 0.5 L of blood was evacuated from the free peritoneal cavity, and the ectopic embryo was found in the ampulla of the right fallopian tube (Figure 3). The EP was successfully removed, and the right fallopian tube was preserved. A histological examination confirmed chorionic villi suggestive of an approximately eighth week ectopic pregnancy. There were no complications during the postoperative course. After the surgery, she was put on progesterone support (200 mg/day intravaginally) and was continued until 12-weeks of gestation. The patient recovered well and was discharged from the hospital on the third postoperative day. She had regular antenatal care appointments, and her pregnancy was uncomplicated. The development of the fetus was normal, and, eventually, at 39 weeks and four days of gestation, the patient gave natural birth to a healthy boy who was 54 cm tall and weighed 3280 g. Postnatal recovery was without any complications, and the patient was discharged on the third postpartum day.

## 3. Discussion

Heterotopic pregnancy is defined as a multiple gestation with one embryo inside the uterus and the other one elsewhere. This condition has become more and more common and relevant because of widespread assisted reproductive technologies (ARTs) and ovarian stimulation for infertility treatment [2,3]. Other risk factors for HP are pelvic inflammatory disease (PID), pelvic surgery, and previous fallopian tube damage or pathology [3]. Our patient did not have any of these risk factors and conceived spontaneously, which makes this case very rare and hard to detect.

In 95% of cases, the EP occurs in the fallopian tube [6], but it can also be found in the cervix, scar from a prior cesarean section, and the interstitial segment of a fallopian tube, ovary, peritoneal, or abdominal cavity [7]. The apparent increase in the incidence of nontubal EPs including HP may be attributed to the higher number of pregnancies after in vitro fertilization treatment [6]. Our case described a case of an EP in the right fallopian tube.

Tal J et al. reported that 70% of all HP cases are diagnosed between five and eight weeks of gestation, 20% between 9 and 10 weeks, and only 10% after the 11th week [2]. The symptoms of HP are nonspecific. HP can be asymptomatic in 24% of cases [4,8]. Abdominal pain is the most frequent symptom of HP, though vaginal bleeding and hypovolemic shock are also common [4,8]. Vaginal bleeding and hypovolemic shock often indicate the rupture of the EP and require urgent treatment. Our patient was admitted to the emergency room complaining of the pain in the abdomen with no other symptoms, which made a differential diagnosis difficult.

The early diagnosis of HP is challenging because a raised serum β-hCG level and an intrauterine embryo seen on US lead one to think about normal pregnancy, and almost no one examines for an EP if the patient is asymptomatic. When an intrauterine embryo is found, it is crucial to inspect the adnexa of the uterus and to record it. The identification of an EP on US has a reported sensitivity and specificity of 71–100% and 41–99%, respectively [9]. Almost half HP cases are detected during emergency laparotomies due to tubal ruptures [4]. Combined serum β-hCG measurement and TVUS improve the diagnostic sensitivity of HP [3]. TVUS has been found to be better in early diagnosis compared to transabdominal US. It detects almost 70% of cases between the fifth and eighth weeks of gestation [10]. In this case, both a serum β-hCG measurement and TVUS were done at the emergency room, and HP was suspected because both embryos were visualized and one of them was outside the uterus.

Treatment possibilities include expectant management, surgical management (either laparoscopy or laparotomy), and sonography-guided embryo aspiration with or without embryo-killing drugs [3,5]. Treatment depends on the patient’s condition, the size and site of an EP, previous pregnancies, the viability of intrauterine and extrauterine gestation, and the expertise of the physicians. Expectant management can be selected in symptom-free patients where the unruptured ectopic embryo has a limited craniocaudal length with no cardiac activity and a decreasing level β-hCG [11]. Though the transabdominal sonographic guided aspiration of an EP has the best maternal outcome and the lowest abortion rate, it should only be chosen as a treatment option when the ectopic gestational sac is clearly visualized [5]. For patients with unstable hemodynamics or with any signs indicating the rupture of extrauterine pregnancy, emergency surgery is strongly recommended [5]. The advantage of surgical treatment is the ability to completely remove an EP, but there might be a higher abortion rate of an IU embryo [5]. In their study, Li J-B et al. found that the total abortion rate was 26.56% in all HP patients and the abortion rate in surgery management group was 25.93% [5]. In our case, an urgent right laparoscopic salpingotomy was chosen due to the free intraperitoneal fluid in the lesser pelvis and the suspicion of the rupture of the EP. The postoperative period was successful with normal growth of the IU embryo. The right fallopian tube was preserved, which is extremely important for young women who will likely want to have more children in the future. About 60–70% of HP cases result in live childbirth with outcomes similar to that of singleton pregnancies [4].

Postoperative luteal phase support in these cases remain controversial. There have yet to be research works that suggest that using progesterone in these conditions would increase the live birth rate. E. Weedin et al. conducted a survey to evaluate the use of progesterone supplementation in non-ART infertility treatments and demonstrated that the empiric use of luteal-phase progesterone supplementation in these treatments is widespread among clinicians even though there is lack of evidence supporting its benefit [12]. The use of progesterone is also prevalent in women with bleeding in early pregnancy who suspect that the bleeding occurs as a result of low progesterone. Nonetheless, many studies have not found a significantly higher incidence of live births when progesterone is prescribed in the first trimester for women with bleeding and no other risk factors as a prophylaxis for miscarriage [13,14]. However, for women who have both bleeding in the first trimester and a history of previous miscarriage, progesterone can have benefit in reducing the risk of miscarrying a fetus [15]. Even though in our case, the patient had no history of miscarries, vaginal progesterone was prescribed for luteal phase support as a safety warranty for this complicated case.

## 4. Conclusions

All pregnant women presenting with abdominal pain or vaginal bleeding should be suspected of heterotopic pregnancy even the conception is spontaneous. Combined serum β-human chorionic gonadotropin measurements and transvaginal ultrasonography are efficient for diagnosing HP. During ultrasound examination, it is very important to check the uterus and the adnexa of the uterus and lesser pelvis. Treatment options include several different methods from observation to surgery and should be chosen depending on the clinical situation. If diagnosed and treated on time, heterotopic pregnancy has favorable outcomes for intrauterine pregnancy and a woman. The need of luteal phase support in this case remain unclear due to a lack of evidence for a good outcome.

## References

[1] DeVoe R.W., Pratt J.H. (1948). Simultaneous intrauterine and extrauterine pregnancy. Am. J. Obstet. Gynecol..

[2] Tal J., Haddad S., Gordon N., Timor-Tritsch I. (1996). Heterotopic pregnancy after ovulation induction and assisted reproductive technologies: A literature review from 1971 to 1993. Fertil. Steril..

[3] Yu Y., Xu W., Xie Z., Huang Q., Li S. (2014). Management and outcome of 25 heterotopic pregnancies in Zhejiang, China. Eur. J. Obstet. Gynecol. Reprod. Biol..

[4] Nabi U., Yousaf A., Ghaffar F., Sajid S., Ahmed M.M.H. (2019). Heterotopic Pregnancy—A Diagnostic Challenge. Six Case Reports and Literature Review. Cureus.

[5] Li J.-B., Kong L.-Z., Yang J.-B., Niu G., Fan L., Huang J.-Z., Chen S.-Q. (2016). Management of Heterotopic Pregnancy: Experience From 1 Tertiary Medical Center. Medicine (Baltimore).

[6] Oron G., Tulandi T. (2013). A pragmatic and evidence-based management of ectopic pregnancy. J. Minim. Invasive Gynecol..

[7] Chukus A., Tirada N., Restrepo R., Reddy N.I. (2015). Uncommon Implantation Sites of Ectopic Pregnancy: Thinking beyond the Complex Adnexal Mass. Radiographics.

[8] Rojansky N., Schenker J.G. (1996). Heterotopic pregnancy and assisted reproduction—An update. J. Assist. Reprod. Genet..

[9] Seffah J.D. (2000). Ultrasonography and ectopic pregnancy—A review. Int. J. Gynaecol. Obstet..

[10] Shah N.H., Shah R.J., Kshirsagar S. (2018). Laparoscopic management of heterotopic pregnancy in an IVF conception. Int. J. Reprod. Contracept. Obstet. Gynecol..

[11] Baxi A., Kaushal M., Karmalkar H.K., Sahu P., Kadhi P., Daval B. (2010). Successful expectant management of tubal heterotopic pregnancy. J. Hum. Reprod. Sci..

[12] Weedin E., Kort J., Quaas A., Baker V.-L., Hansen R.-K. (2016). Progesterone supplementation for luteal phase support in non-assisted reproductive technology treatments-prevalence of use and practice patterns among infertility specialists. Fertility and Sterility.

[13] Haas D.M., Ramsey P.S. (2008). Progestogen for preventing miscarriage. Cochrane Database Syst. Rev..

[14] Coomarasamy A., Devall A.J., Cheed V., Harb H., Middleton L.-J., Williams H., Roberts T., Goranitis I., Ahmed A., Gallos I.D. (2019). A Randomized Trial of Progesterone in Women with Bleeding in Early Pregnancy. N. Engl. J. Med..

[15] Haas D.M., Hathaway T.J., Ramsey P.S. (2019). Progestogen for preventing miscarriage in women with recurrent miscarriage of unclear etiology. Cochrane Database Syst. Rev..

